# Supplementary material for: Multi‐dataset identification of innovative feature genes and molecular mechanisms in keratoconus
Source: J Cell Mol Med. 2024 Sep 19;28(18):e70079. doi: 10.1111/jcmm.70079 (PMC11412914; doi:10.1111/jcmm.70079)
Supplement: Supplementary file 2 — Material S2. [file JCMM-28-e70079-s002.zip › 7.GSEA analysis/1.ARL11/KEGG_GALACTOSE_METABOLISM.html]

Details for gene set KEGG\_GALACTOSE\_METABOLISM[GSEA]

|  || Dataset | ARL11.group.cls#h\_versus\_l |
| Phenotype | group.cls#h\_versus\_l |
| Upregulated in class | h |
| GeneSet | KEGG\_GALACTOSE\_METABOLISM |
| Enrichment Score (ES) | 0.31528646 |
| Normalized Enrichment Score (NES) | 0.9676565 |
| Nominal p-value | 0.5 |
| FDR q-value | 1.0 |
| FWER p-Value | 1.0 |
Table: GSEA Results Summary

  

Fig 1: Enrichment plot: KEGG\_GALACTOSE\_METABOLISM      
 Profile of the Running ES Score & Positions of GeneSet Members on the Rank Ordered List

  

| SYMBOL | TITLE | RANK IN GENE LIST | RANK METRIC SCORE | RUNNING ES | CORE ENRICHMENT || 1 | HK3 | na | 938 | 0.397 | 0.1248 | Yes |
| 2 | LCT | na | 1127 | 0.373 | 0.2621 | Yes |
| 3 | LALBA | na | 2679 | 0.256 | 0.3153 | Yes |
| 4 | B4GALT2 | na | 8950 | 0.076 | 0.1635 | No |
| 5 | PFKP | na | 9473 | 0.068 | 0.1744 | No |
| 6 | GANC | na | 9905 | 0.061 | 0.1854 | No |
| 7 | GALE | na | 10429 | 0.055 | 0.1912 | No |
| 8 | GALK2 | na | 13649 | 0.022 | 0.1067 | No |
| 9 | GAA | na | 13707 | 0.021 | 0.1131 | No |
| 10 | GALK1 | na | 13765 | 0.021 | 0.1194 | No |
| 11 | PFKM | na | 15872 | 0.005 | 0.0605 | No |
| 12 | PGM2 | na | 17963 | -0.010 | 0.0041 | No |
| 13 | GLB1 | na | 18269 | -0.012 | -0.0000 | No |
| 14 | PFKL | na | 19009 | -0.017 | -0.0149 | No |
| 15 | HK1 | na | 19947 | -0.023 | -0.0329 | No |
| 16 | UGP2 | na | 20718 | -0.029 | -0.0440 | No |
| 17 | B4GALT1 | na | 21286 | -0.033 | -0.0475 | No |
| 18 | GLA | na | 23413 | -0.055 | -0.0878 | No |
| 19 | GALT | na | 24828 | -0.074 | -0.1004 | No |
| 20 | PGM1 | na | 25044 | -0.077 | -0.0773 | No |
| 21 | GCK | na | 25358 | -0.081 | -0.0554 | No |
| 22 | G6PC2 | na | 25991 | -0.090 | -0.0391 | No |
| 23 | HK2 | na | 26843 | -0.105 | -0.0235 | No |
| 24 | AKR1B1 | na | 30604 | -0.208 | -0.0524 | No |
| 25 | MGAM | na | 34174 | -0.448 | 0.0160 | No |
Table: GSEA details [plain text format]

  

Fig 2: KEGG\_GALACTOSE\_METABOLISM      
 Blue-Pink O' Gram in the Space of the Analyzed GeneSet

  

Fig 3: KEGG\_GALACTOSE\_METABOLISM: Random ES distribution      
 Gene set null distribution of ES for **KEGG\_GALACTOSE\_METABOLISM**
